# Commentary: Unmapped waters—navigating a sea of institutional preferences in cardiogenic shock management

**DOI:** 10.1016/j.xjon.2020.03.004

**Published:** 2020-04-18

**Authors:** Michael Salna, Hiroo Takayama

**Affiliations:** Division of Cardiac, Thoracic, and Vascular Surgery, Department of Surgery, Columbia University Irving Medical Center, New York, NY

**Figure:**
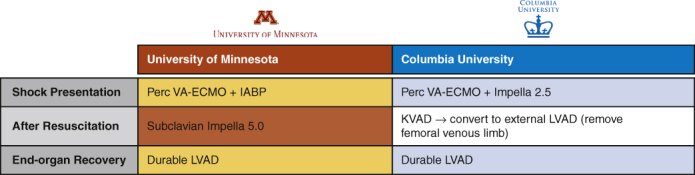
Institutional differences in mechanical circulatory support for the same patient.

**Central Message**

Patients in cardiogenic shock benefit from multidisciplinary shock team evaluation and early VA-ECMO. Transition to an Impella can reduce ECMO complications in those not yet ready for weaning.

See Article page 29.

Cardiogenic shock presents one of the most challenging pathologies to manage, largely due to an absence of guidelines. There are 3 significant factors preventing the development of an expert consensus, and, consequently, evidence-based guidelines. These culminate in a complex interplay between (1) a heterogeneity of surgical expertise and preference, (2) local resource availability, and (3) a patient's clinical picture.

Qi and colleagues^1^ attempt to shed some light on this dilemma through a decision-making framework in progressing the care of patients receiving venoarterial extracorporeal membrane oxygenation (VA-ECMO) at the University of Minnesota. They advocate for “on-time” initiation of VA-ECMO in refractory cardiogenic shock—ECMO before multisystem organ failure develops—as well as a rapid transition to other, less-morbid, forms of mechanical circulatory support (MCS), namely the Impella 5.0 (Abiomed, Danvers, Mass). This early ECMO strategy not only optimizes outcomes but also facilitates their bridging strategy to a subclavian Impella for those requiring longer-term support. Qi and colleagues outline how to prepare for subclavian Impella 5.0 transition, including assessing subclavian size with ultrasound early to determine suitability as well as their exclusion criteria of a subclavian diameter <7.0 mm, the continued need for an oxygenator, the presence of left ventricular (LV) thrombus, more than mild aortic insufficiency, or severe refractory right ventricular dysfunction.

**Figure 1:**
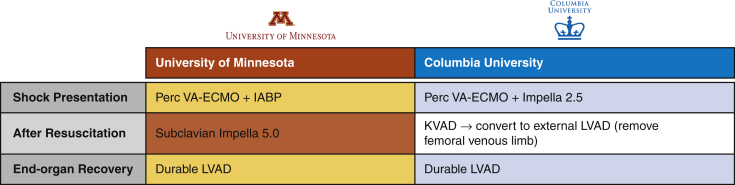
Institutional differences in mechanical circulatory support treatment algorithms for the same patient. *Perc*, Percutaneous; *VA-ECMO*, venoarterial extracorporeal membrane oxygenation; *IABP*, intra-aortic ballon pump; *KVAD*, femoral venous cannula with direct left ventricular vent to axillary arterial return; *LVAD*, left ventricular assist device.

This decision-making framework, however, is simply one institutional, anecdotal, evidence-based opinion. And therein lies the primary challenge of obtaining clinical equipoise: every program respects and learns from one another but has strong reasons to continue with their current strategy. For example, Figure 1 illustrates how the 32-year-old patient in the report's vignette would have had been treated in northern Manhattan. At shock presentation, we *believe* LV offloading is better accomplished with the Impella 2.5, given its superior support to an intra-aortic balloon pump and its independence of intrinsic LV function,^2^ and we *prefer* the “KVAD” configuration (ie, femoral venous cannula with direct LV vent to axillary arterial return) over any temporary LVAD after resuscitation, as it permits gradual right ventricular and pulmonary support weaning.^3^ Again, these are merely the *beliefs* and *preferences* of our institution.

At our institution, we agree with the early initiation of MCS and have found that VA-ECMO tends to be a more supportive initial therapy over the Impella.^4^ As Qi and colleagues point out, peripheral VA-ECMO is associated with considerable morbidity, including bleeding, thrombosis, infection, and limb ischemia. These costs, however, may be reasonable to pay for the benefit of the most effective peripheral modality for systemic perfusion and oxygenation. The question then arises—do the economic costs of regularly transitioning to a subclavian Impella outweigh the risks of several more days on ECMO? We do not know the answer, but perhaps it is worth pursuing.

The authors conclude by rightly advocating for the widespread use of multidisciplinary shock teams to identify cardiogenic shock early, thereby permitting the initiation of more aggressive treatments with the hopes of avoiding MCS altogether. This early identification strategy may hold the most promise for the future, as patients cannot suffer the consequences of VA-ECMO if they are able to avoid it in the first place.
